# The Social Evil1Delivered by invitation at the Commencement Exercises before the Alumni Association of the Medical Department of the University of Buffalo, May 31, 1906.

**Published:** 1906-12

**Authors:** Denslow Lewis

**Affiliations:** Chicago, Illinois., Chairman of the Section on Hygiene and Sanitary Science of the American Medical Association. 92 State Street


### BUFFALO MEDICAL JOURNAL.

VOL. LXII. DECEMBER, 1906. No. 5

### ORIGINAL COMMUNICATIONS.

### The Social Evil.[1]

BY DENSLOW LEWIS, M. D., Chicago, Illinois.
Chairman of the Section on Hygiene and Sanitary Science of the American Medical Association.

INDEX THROUGH

AN occasion like this is usually improved to offer congratulations to the new members of the craft who have served their apprenticeship and are now joyfully admitted with us to the rank of master workmen. It is also customary for some one to speak of the high ideals of the medical profession, to give perfunctory advice, and to indulge in words of eulogy and encouragement. It is certainly a glad day in any man's life when fruition comes at last as the result of honest labor.

I look back now to a day nearly thirty years ago when I was found worthy to take my place with the others, to do what I might to bind up the wounds that bleed, and above all things, by judicious endeavor, to try to learn how a wise and practical prophylaxis might limit disease and increase the usefulness and happiness of the human race. My struggles will not be yours. Honest men have worked faithfully and well for humanity and, although in many instances even their names are now forgotten, the imprint they have left on medical progress and the incentive they have given to modern thought attest the usefulness of their lives, and form a more substantial reward than title, worldly honor, or the accumulation of property. Medical science has advanced, and the new generation begins where the workers of the present leave off.

It is, I think, interesting to observe that the profession realises now more than ever that its members have other things to do than wield the knife or roll the pill. It is a happy sign of the times when medical men understand that they must study sociology, that they must appreciate economic conditions, that they must face the facts and know life as it is, and not as their wishes would have it be. A great step in advance is taken

1. Delivered by invitation at the Commencement Exercises before the Alumni Association of the Medical Department of the University of Buffalo, May 31, 1906.

when we really believe that our way, while it seems to us to be the best, is not the only way; that our creed may not be the only true creed; that our standard of right and wrong and justice and truth may be only relatively correct. Above all things it is most fortunate for humanity and most propitious for real advancement, when we begin to discern that precedent is not authority, that human judgment may err, that civilisation advances, and that our knowledge of the truth is progressive.[1]

It is only of late years that we have learned all this. We have of course admitted it in a very general way, as every man who thinks must do. We have been hampered in the study of many of our most serious conditions by a false modesty and a maudlin sentimentality which prevented us from looking facts well in the face. We have thought, as at least our actions would seem to indicate, that ignorance meant innocence, and that certain conditions of our society were so deplorable and disgusting that it were shameful even to admit their existence.[2] And yet we knew they existed; we knew the illness, the suffering of body and the distress of mind they caused. We tried to suppress them, but they would not down. We tried to ignore them, but their effects were so manifest that we realised the folly of closing our eyes and saying we did not see. At last we have come to our senses, and our better judgment prevails. We know there must be a reason for every event and a cause for every effect. We know there must be an incentive for every action and we understand now, in a most practical manner, that our only hope of effecting radical improvement consists first of all in a true understanding of the relationship between cause and effect and in realising that a knowledge of all the facts is of the first importance.[3]

In this liberalising spirit of inquiry, I purpose to offer for your consideration some of the facts, as I understand them, regarding what is known as the social evil. It is, I believe, the first time in America that this topic has been the subject of an address of this kind. Education and culture cause increased responsibility. It is a happy augury of the future that intelligent men and women throughout the country wish to devote serious thought to this most momentous problem that pertains to our civilisation. Their work will bear fruit. They cannot study this great question of the ages without some practical benefit. They have taken the first step, they cast aside all prudery, they investigate with candor and deliberation this condition of our society and the direful effects that result from it, they know the folly of concealment, they wish to show the danger. The value of publicity is acknowledged.

With this preface which, if you please, may serve as an apology for my topic, if it is thought that an apology is necessary, I state that next in importance to the instinct that demands the preservation of the individual is the instinct which determines the perpetuation of the species. This is a simple thing to say and a fact which is universally acknowledged. There is nothing disgusting about this. It shows to my mind a wonderful provision for the continuance of the race. This sexual instinct is the basis of love, for without its presence the platonic affection that exists between man and woman is only friendship. This need not be a startling declaration. I do not mean that the man who loves a pure young woman wishes to take an unfair advantage of her affection for him. I do not underestimate in the young woman the value of culture, education, and refinement, but I do maintain that the beauty of face and form, given in their perfection to women only during the years when childbearing is possible, indicate that her chief mission is that of the mother and that her most exalted station is that of the wife who makes possible the home which is the cornerstone of our society.

This matter of the sexual instinct must be studied very carefully and most thoroughly. It is the impelling force in our nature. It demands satisfaction. Its consideration may not be dismissed with a shrug of the shoulders, neither is it just or wise to call it nasty. It must be remembered it means home and children and happiness. It is the noblest function of which any man or woman is capable. It is the keynote of a harmonious community. It exists and it must exist or there is no future to the race, no happiness to human life, no guarantee of safety to the commonwealth.[4]

With the age of puberty comes the awakening of the sexual instinct. It is usually more accentuated in the boy than in the girl. It makes itself evident in an unmistakable manner, and unless the child is told about it, there is danger of masturbation, unnatural practices, or premature intercourse which may prove disastrous in many ways. As the child grows older, as adult life is reached and indeed during the greater part of every healthy life, the sexual instinct exerts itself in a dominating manner which, of course, is to be expected and which we must admit is desirable when we realise that were this not the case the race would be in danger of extinction. It is due to this sexual instinct that the social evil exists. It is chiefly due to the masterful influence of this instinct in the male that prostitution is possible.

I wish here to discriminate in regard to the use of a few expressions. When we say "evil," we must realise that there is a relative meaning to the word. If it is an evil for a man to cohabit with a public woman, it is preferable to have him do this rather than to attempt to seduce our daughters or alienate the affections of our wives. It is infinitely preferable for him to buy the goods where they are for sale than to take advantage of the ignorance, the poverty, or the credulity of the young working girl. The word prostitute has been defined as the woman who sells her body lewdly and indiscriminately for money. We sometimes hear of the male prostitute. He exists, but he is not the man who is the customer of the prostitute. He offers his services for money as she does hers, and his patrons, like himself, are often degenerates or perverts who indulge in unnatural practices.

Now what are the facts regarding prostitution as we know it? My friend, Valentine, of New York, claims that hereditary degeneration obtains, producing a reversal to primary conditions when polyandry existed.[5] I presume that is true in some instances, but my observation teaches me that the unfortunate young girl is apt to take up this calling as a means of livelihood when all other avenues of employment are barred to her. If she has been forced to steal and has served in the reform school or bridewell, it is almost impossible for her to secure decent employment. No one wants the thief even if she be virtuous, or if extenuating circumstances exist in her case. We know the fate of the indiscreet girl who is found out. There is no forgiveness for her. We know what happens when the unmarried girl becomes pregnant. She is driven from home or from her place of employment. There is no excuse for her. If a criminal abortion is performed, the inhumanity of those she considered her friends often induces a spirit of recklessness which lands her in the house of prostitution. If she has an illegitimate child, the procuress often awaits her departure from the maternity hospital. Deserted by her seducer, unable to find employment among those who know of her and her disgrace, forsaken, destitute, and disheartened, the Salvation Army or the house of prostitution is practically her only refuge.

Prostitutes are often debauched and degraded, the vilest of women. I cannot help but remember that there was a time when each one was a pure young child. In rare instances I know the sexual instinct is so strong in a girl that she forgets her caution. In most instances I know the fault is the man's. He has insinuated himself into her affection, he has imposed upon her credulity, he has taken advantage of her ignorance or her necessity, he has not realised that in honor he should be the protector of the young girl if need be even against herself.

I speak of these things, which I know to be facts, because I am indignant when I hear so much talk about these depraved women. I know they are often depraved, but I know men make them so. If man did not insist, if our civilisation, our education, and our religion could teach him self-control, the prostitute would not be. So it is as unjust as it is cruel for us to condemn these women for doing what men make them do. If prostitution is to cease let us stop the demand by teaching our men and boys the possibility of continence, the advisability of chastity, the dignity of virility. There will be no supply unless there is a demand.

And women must view this subject in the light of justice. When we read daily of men of prominence who are shown to be defaulters, when the life of shame of many a man of exalted station is exposed, is it not time that our women should look in pity on the girl whose necessities have made her compliant? Is it not fair that the unfortunate ones should in charity assist the unfortunate? Who can tell what might have been the outcome to any woman under similar conditions?

It is safe to assert that prostitution will not cease during our lifetime or for several generations to come. It will, in my opinion, last until educational methods teach the man his duty and economic conditions render the girl self-supporting. In the meantime the prostitute exists, we have seen how and why, and the question is, "What shall we do with her?" I answer first, she is a woman and a citizen. She can hold property, she can do theoretically at least, very much as other women. Knowing how she has been forced into her present life and knowing further her ultimate fate in a few short years, I can not bring myself to spit upon her, to push her deeper down into the mire. I realise also, under our present imperfect and incomplete system of civilisation, the great good that she really does in every community. This perhaps seems incredible,—almost incomprehensible. But note what Lecky, the great English moralist, says of the prostitute: "The supreme type of vice, she is ultimately the most efficient guardian of virtue. But for her happy homes would be polluted, abortion and infanticide would increase, unnatural and most harmful practices would abound."[6]

Here are startling thoughts unless we have studied the subject carefully and in the spirit of justice. To most women the prostitute seems so degraded that her presence even in the public street is resented. And yet she may indirectly have saved the daughter from ruin; she may have saved the wife from divorce or abandonment. She is unfortunate, a derelict on life's ocean. Save her if you can, give her due credit for what good she does, remember she is what she is, because some man has made her so, remember she is after all a woman.

The police regulations that apply to prostitution, like most municipal regulations, are a compromise. We wish there were no prostitutes, but we know there must be until the demand for them shall cease, when our educational measures become truly effective and our religion becomes an active force in daily life instead of chiefly a belief in a creed. Under existing conditions it would seem to be wise not to try to crush out prostitution, for history shows such an attempt is impossible of execution, and our knowledge of human nature would seem to indicate that there are, after all, more serious evils to be feared. Common sense apparently indicates that the most judicious thing to do is to prevent outrage and crime and to try to save the young. For this reason segregation is usually advisable. The man in quest of the prostitute will find her. There is no reason why in so doing he should be exposed to theft or assault. The prostitute has the right to live but she may not solicit nor become, or maintain a nuisance. It is better to have her live in a district of her own than in the neighborhood of our schools or our homes. Our boys will find her if they determine to do so, but it is better for them to hunt her out knowingly and willingly than to be lured to her abode by a street acquaintance, or meet her by chance late at night when perhaps undue indulgence in alcohol may have impaired the judgment. In a word, she should be where she may be found, but she should not herself do the seeking.

Investigation in New York and other large cities has shown a deplorable state of affairs in relation to the police and their methods. The frequent raids, the cry of graft, the exposure of connivance are humilating to our sense of decency and serve to demonstrate the need of a true surveillance which shall not be an unjust oppression. Nuisances must be abated, crime must be suppressed, order must be maintained. There is, however, no justification for bribery, blackmail, or unfair intimidation. Within her own limits, the prostitute has the right to live. Ostentatious solicitation should be prohibited, obscenity should be checked, decorum should be demanded. The outrages committed by the police in the name of the law are not justifiable and are especially reprehensible when the victims are unfortunate women of this class.

There is another phase to this question which is of special interest to medical men and which has forced the attention of the public to a practical consideration of prostitution. I refer to the venereal diseases. Modern appreciation of these diseases shows that potentially they are far-reaching in their effects and most disastrous in their results. They cause a greater morbidity among adults than all other diseases combined. And then there is this sad fact which must be stated. The innocent becomes infected, often unwittingly, through ignorance. The young wife becomes an invalid for life or a candidate for the operating table. The babe is born blind; it is born sickly or deformed and often succumbs early in infancy. Young children acquire these diseases innocently. The danger is imminent and threatens everyone.

Now I maintain that health is of the first importance and that all disease is to be prevented as far as possible. There are men and women who think otherwise in reference to the venereal diseases. They claim that the man who cohabits with a prostitute gets no more than his deserts if he is infected. If this statement is true, our duty is plain. We must encourage the spread of venereal disease to make sure that no transgressor fails to receive his just punishment. The most distressing circumstance in this connection is the infection of the innocent. If the man deserves what he gets, surely his young wife and his children should not suffer. It is this dissemination of the disease among the innocent, perhaps more than any other single element in its consideration, that has induced, within the past few years, a concerted effort in Germany, France, and other European countries which has for its object the restriction of the venereal diseases.

In the United States this effort took form in February, 1905, when there was organized the American Society of Sanitary and Moral Prophylaxis. In the opening address the president of the society, Dr. Prince A. Morrow, of New York, said; "A free discussion is, of course, an essential preliminary to any well-considered action, especially when such action proposes to deal with what is confessedly the most difficult of all problems of social hygiene."[7] This seems reasonable and yet the student of the subject knows what a great advance even in professional opinion is indicated by the acceptance of this statement. There has been in the past such an intense feeling of loathing against prostitution and venereal diseases that the mere mention of them even in medical societies was considered outrageous. It was said we must not "recognise" vice, traffic with crime, temporise with sin, or consort with the Lords of Hell—whatever that may be. Now, I hold that recognition does not mean sanction or legal endorsement. We recognise murder, arson, poverty, imbecility, degeneracy, and other distressing conditions of modern society; we do not approve of any of them. We wish they did not exist, but it is absurd to deny their existence.

I will not now go into detail regarding the different attempts that have been made in the past to crush out prostitution and venereal disease. Occasionally the methods used have been effectual for a time, but in general it may be said that the results obtained have not been satisfactory. For that reason the attempt now being made is along different lines. It consists first of all in the education of the people which is a matter of the highest importance. Wherever men are thrown together, in factories, in church clubs, in labor unions, in secret society lodges, the teacher will go forth to tell the truth. In France a dramatist named Brieux has produced plays which show how venereal disease may extend. In all European countries pamphlets are being written and circulated broadcast. Our American Society has just issued its first educational pamphlet, and others will follow. In addition to diffusing the knowledge we already possess will come the very earnest study of all economic and medico-sociologic factors in the etiology of the subject. There will come also, as is now advocated in France, increased provision for the gratuitous treatment of venereal diseases as well as a refuge for the infected woman, so that in her effort to live she may not further convey the disease.

In a word, common sense will prevail. There will be no spasmodic attempt to crush out prostitution, no mock heroics which indicate the Pharisee. There will be a true humanitarianism and a real Christianity. There will be work done, honest and effective work. It is not enough to say, as does my distinguished friend, Howard Kelly, that the answer to this momentous question is "definite faith in God, a faith which leans upon Him at all times for grace and strength to do that which we otherwise could not do." Neither is it good sense to sum up the whole matter as he has done recently, as follows: "My only hope lies solely in God and in prevailing on men to look to Him for grace and strength to do that which they cannot of themselves accomplish."[8]

In no way would I belittle the value of religion, for without hope in God any work of man is of little avail. Across the ages the great figure of the Nazarene stands forth as the exponent of the doctrine of love which makes all men brothers. Let us have faith and hope in God, but let us do our work unfalteringly. Let us give even the prostitute a square deal, save her if we can, but at all events prevent her from spreading disease. Let us be honest and sincere, look facts in the face, reason, investigate, and above all things tell what we already know. Thus, in my opinion, may we practically and definitely help to solve this momentous problem.

These few thoughts relating to the social evil I beg to submit to you. I do not pretend to say I am absolutely right but at least I think I am right and my statements are not made carelessly or without consideration and observation. I know the old methods are ineffectual and everybody who knows about them admits this. The profession now starts anew to dispel the darkness of ignorance by bearing aloft the light of truth. We look to a rational education as our supreme hope. We believe in a religion of deeds, a civilisation which protects the interests of the humblest citizen. We think it is possible to teach men the meanng of honor and to give women a knowledge of charity. We know it is unjust to condemn the young girl when there has been no warning of the danger that confronts her. We believe in adequate wages for the girl so that she may live without selling herself. These elements in our civilisation must be investigated. By a persistent and intelligent effort to strike at the cause, the social evil must be controlled and venereal infection must be eradicated.

92 State Street.

REFERENCES.

1. Denslow Lewis: Ignorance as a Cause of Disease and Disaster; Ills. Med. Journ., Jan., 1906.
2. Denslow Lewis: The Gynecologic Consideration of the Sexual Act.
3. Denslow Lewis: Knowledge as a Factor in Venereal Prophylaxis; Am. Journ. of Dermatology, Feb., 1906.
4. Denslow Lewis: The Need of Publicity Regarding Venereal Diseases; Medical Record, 1906.
5. Denslow Lewis: The Limitation of the Venereal Diseases; Medico-Legal Journal, June and Sept., 1903.
6. William E. H. Lecky: The History of European Morals from Augustus to Charlemagne.
7. Prince A. Morrow: The Society of Sanitary and Moral Prophylaxis, its Objects and Aims; American Medicine, Feb. 25, 1905.
8. Howard A. Kelly: The Regulation of Prostitution; Journ. Am. Med. Association, Feb. 10, 1906.

